# Non-malignant pathological results from CT-guided biopsy for pulmonary nodules: a predictive model for identifying false-negative results

**DOI:** 10.1186/s13019-024-02898-2

**Published:** 2024-06-26

**Authors:** Xu-Zhou Wang, Jing-Ya Wang, Tao Meng, Yi-Bing Shi, Jin-Jun Sun

**Affiliations:** 1 Department of Radiology, The First Affiliated Hospital of Soochow University, Suzhou, China; 2 Department of Radiology, Xuzhou Central Hospital, Xuzhou, China; 3 Department of Nuclear Medicine, Xuzhou Central Hospital, Xuzhou, China

**Keywords:** Computed tomography, Biopsy, Non-malignant, Predictive model

## Abstract

**Background:**

Computed tomography (CT)-guided biopsy (CTB) procedures are commonly used to aid in the diagnosis of pulmonary nodules (PNs). When CTB findings indicate a non-malignant lesion, it is critical to correctly determine false-negative results. Therefore, the current study was designed to construct a predictive model for predicting false-negative cases among patients receiving CTB for PNs who receive non-malignant results.

**Materials and methods:**

From January 2016 to December 2020, consecutive patients from two centers who received CTB-based non-malignant pathology results while undergoing evaluation for PNs were examined retrospectively. A training cohort was used to discover characteristics that predicted false negative results, allowing the development of a predictive model. The remaining patients were used to establish a testing cohort that served to validate predictive model accuracy.

**Results:**

The training cohort included 102 patients with PNs who showed non-malignant pathology results based on CTB. Each patient underwent CTB for a single nodule. Among these patients, 85 and 17 patients, respectively, showed true negative and false negative PNs. Through univariate and multivariate analyses, higher standardized maximum uptake values (SUV_max_, *P* = 0.001) and CTB-based findings of suspected malignant cells (*P* = 0.043) were identified as being predictive of false negative results. Following that, these two predictors were combined to produce a predictive model. The model achieved an area under the receiver operating characteristic curve (AUC) of 0.945. Furthermore, it demonstrated sensitivity and specificity values of 88.2% and 87.1% respectively. The testing cohort included 62 patients, each of whom had a single PN. When the developed model was used to evaluate this testing cohort, this yielded an AUC value of 0.851.

**Conclusions:**

In patients with PNs, the predictive model developed herein demonstrated good diagnostic effectiveness for identifying false-negative CTB-based non-malignant pathology data.

## Background

Computed tomography (CT)-guided biopsy (CTB) is a minimally invasive approach that is commonly used to diagnose tumors and tumor-like lesions. Although CTB-based methods are often used for identifying pulmonary nodules (PNs) and can achieve diagnostic accuracy rates ranging from 90 to 96% [1–3], the diagnostic yield for these procedures usually varies from just 42–68% [1, 3]. This is because only specific benign findings of malignant pathology results can be considered as a definitive diagnosis while performing CTB [1–3]. In comparison, a definitive diagnosis cannot be established when CTB produces a suspected malignant result or a non-specific benign result, such as one associated with fibrosis, granulomatous inflammation, or chronic inflammation [4]. Under these circumstances, diagnoses must generally be made based on repeated CTB, careful follow-up, or the resection of the lesion of concern [5].

An estimated 83.6–89.6% of CTB-based non-malignant cases are believed to correspond to truly benign lesions [4–6]. Therefore, it is crucial to be able to precisely detect and recognize cases where negative results are inaccurate. Previous studies have indicated that CTB-based granulomatous inflammation is frequently indicative of true-negative outcomes [4–6]. However, the diagnostic utility of CTB-based granulomatous inflammation in isolation is reportedly quite limited, with sensitivity values ranging from 40.1 to 54.8% [5, 6]. Therefore, it is necessary to develop a predictive model that includes numerous important factors to provide a more thorough understanding of cases with CTB-based non-malignant findings.

The objective of this study was to detect the predictors that can accurately identify cases when patients with PN have false-negative results from CTB for non-malignant results. The findings from this study will be used to construct a predictive model.

## Methods

This retrospective analysis was approved by the Ethics Committees of The First Affiliated Hospital of Soochow University and Xuzhou Central Hospital. The requirement for written informed consent was waived.

### Study design

**Fig. 1:**
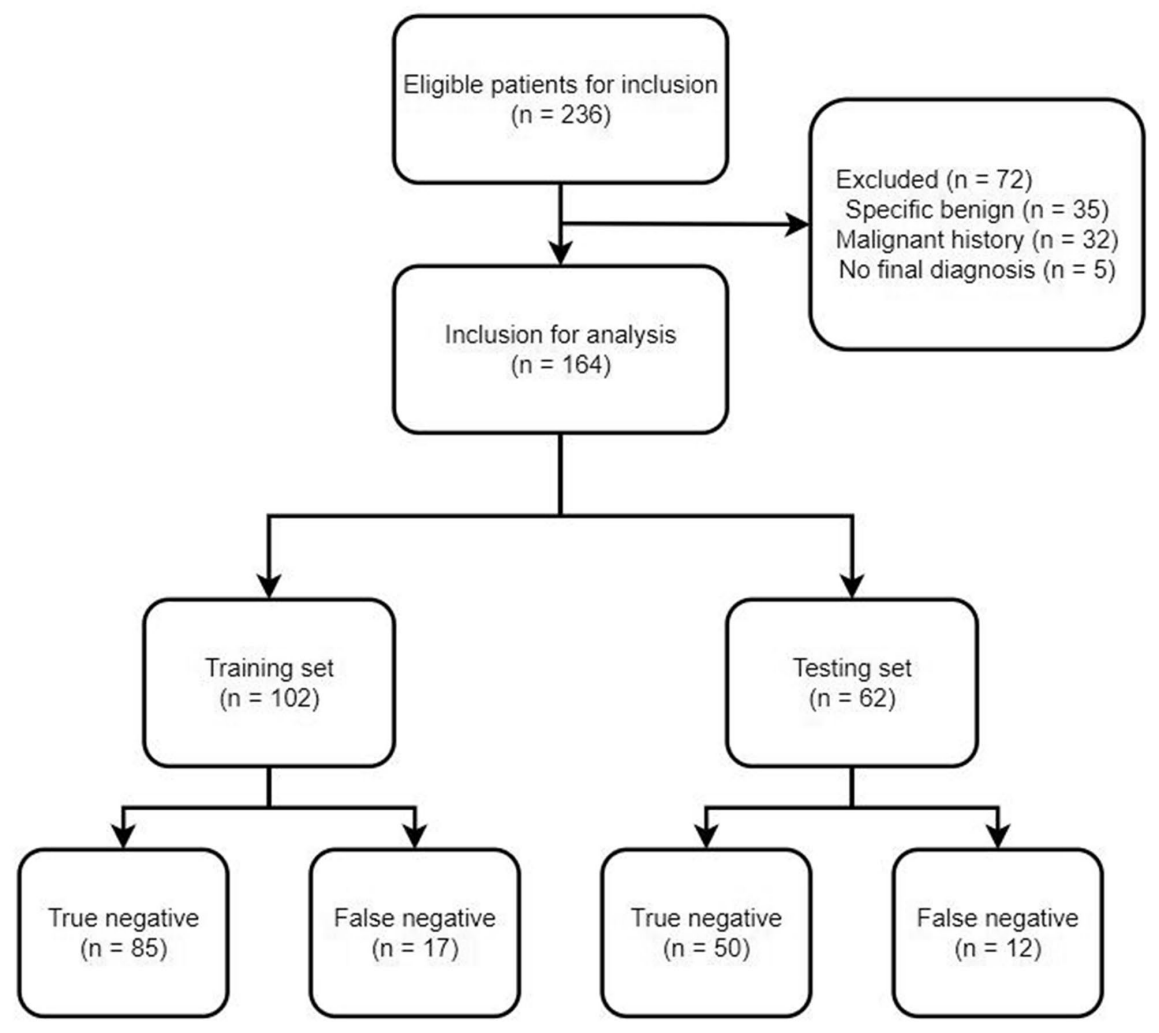
The flowchart of this study

This study enrolled consecutive patients with PNs from two hospitals who obtained CTB-based non-malignant pathological results between January 2016 and December 2020. The training cohort included patients from The First Affiliated Hospital of Soochow University, whereas the testing cohort included patients from Xuzhou Central Hospital (Fig. 1).

Patients who were eligible for inclusion were: (a) individuals with PNs, (b) individuals for whom CTB yielded non-malignant pathological results, and (c) patients who had undergone positron emission tomography (PET)/CT examination within 2 weeks period around initial CTB. Patients were excluded if they: (a) had a history of malignancy, (b) received CTB findings indicative of a specific benign result, or (c) did not have a final diagnosis available for the target PN.

### CTB procedure

All patients underwent CTB with a 16-row CT instrument (Philips, Cleveland, OH, USA) with a co-axial technique. The respective voltage, current, and thickness settings were 120 kV, 150 mA/s, and 1 mm. The patients were positioned according to the location of the target nodule, and the selection of the needle pathway was carried out by an interventional radiologist with more than 5 years of experience based on the results of CT scan. Following confirmation of the puncture site, the lung parenchyma was punctured using a 17G co-axial introducer (DuoSmart™, Modena, Italy) under local anesthesia (2% lidocaine, 5 mL). A follow-up CT scan was then carried out to confirm the location of the needle tip. When the tip was in contact with the target PN, an 18G inner semi-automatic core needle (Wego™, Weihai, China) was inserted via the co-axial introducer and used to collect samples from this nodule. At least 2 samples per PN were obtained and placed in a 10% formaldehyde solution until they were evaluated for pathological analysis. Postoperative CT scans were conducted following removal of the needle to identify potential complications.

### PET/CT imaging

PET/CT was conducted with a Gemini TF PET/CT system (Philips). The 2 centers used the same type of PET/CT device. Each patient was fasted for at least 6 h before examination. The intravenous injection of 3.7MBq/kg of ^18^F-FDG was conducted and each patient waited for 1 h before examination. The CT scan parameters were: voltage 120 kV, current 300 mA, pitch 0.829, collimation 64 × 0.625 mm, rotation time 0.5 s, and reconstruction thickness 5.0 mm. For PET scan, we used the 3-dimension model from the skull base to the middle of the thigh, with the scan time of 1.5 min per bed. All images data were transferred to Philips EBW 3.0 Workstations to reconstruct PET, CT and PET/CT fusion images. The standardized maximum uptake values (SUV_max_) of each PN was automatically calculated.

### Definitions

PNs were defined as ovoid or round lesions ≤ 3 cm in diameter and fully encircled by the pulmonary parenchyma without any related abnormal findings [7]. CTB-based non-malignant pathology outcomes included both non-specific benign and suspected malignant findings. Specific benign results were assigned to lesions that were associated with benign tumors or specific infectious disease, such as bacteria, mycobacteria, or fungi that may explain the observed radiological findings [5, 8]. Non-specific benign results included cases where benign pathological signs, such as fibrosis or inflammatory cells were detected, but these results were not enough to provide a specific diagnosis [5]. Suspected malignant results were defined as atypical cells which were suspected to be indicative of malignancy, however, the findings did not have sufficent evidence for malignant [5].

**Fig. 2:**
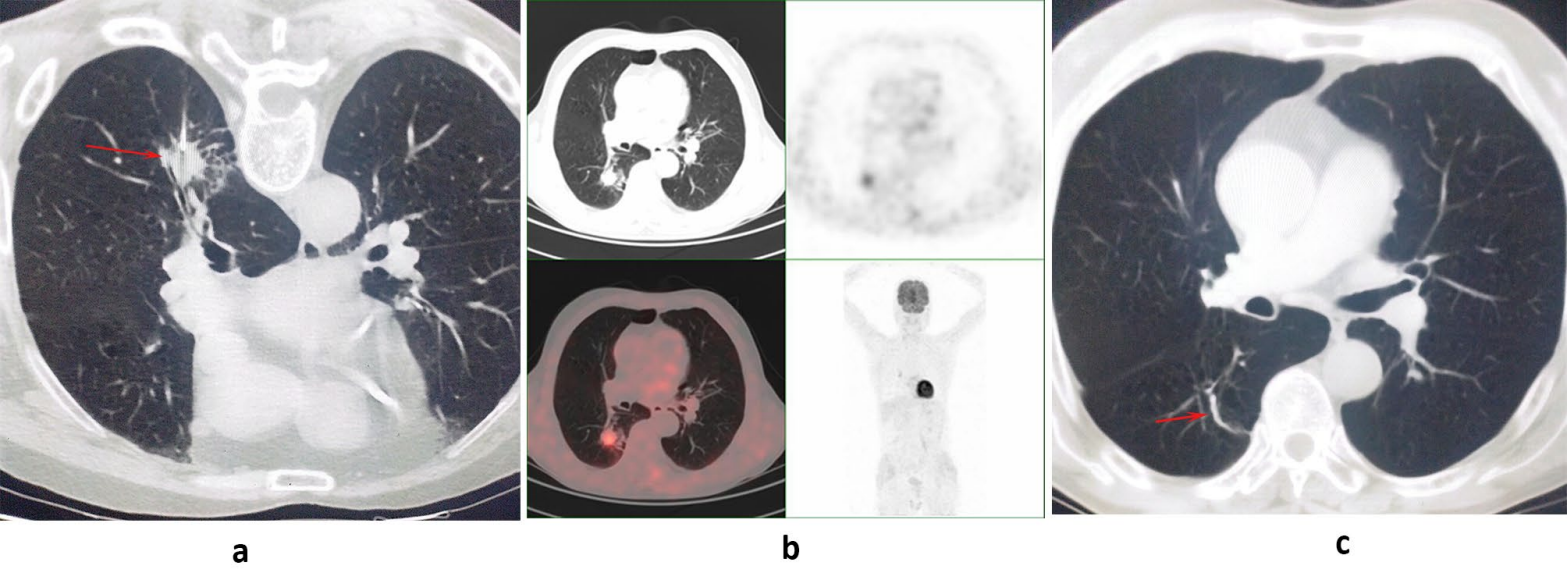
(**a**) A PN (arrow) was biopsied under the CT guidance, and the CTB indicated the diagnostic result was chronic inflammation. (**b**) This patient underwent PET/CT examination after CTB, and the SUV_max_ value was 2.8. (**c**) The PN significantly decreased (arrow) 6 months after CTB, and it indicated a true negative result

Final diagnoses for malignant PNs were made based on either surgical resection or CT-based malignant findings. Final diagnoses for benign PNs were confirmed through surgical resection, CTB-based specific benign results, a > 20% reduction in node diameter (Fig. 2), or stable size (unchanged or < 20% reduction) over at least 1 year without any anticancer treatment [1, 9, 10].

### Statistical analyses

Means ± standard deviations or medians (Q1; Q3) were used to report normally distributed and skewed continuous data, respectively. Student’s t-tests and Mann-Whitney U tests were used to compare the data. Categorical data were compared with the χ^2^ test or Fisher’s exact test. Logistic regression analyses were employed to identify those factors capable of predicting false-negative results, with multivariate analyses incorporating all variables significant (*P* < 0.1) in univariate analyses. The significat factors in the multivariate analysis were used to develop a predictive model and nomogram. Area under the receiver operating characteristic (ROC) curve (AUC) values were compared with the DeLong test. R 4.1.2 and SPSS 26.0 were used for all statistical comparisons.

## Results

### Training cohort

The training cohort included 102 patients with PNs who received CTB-based non-malignant pathological results, with each patient having been evaluated for a single PN. All PNs were solid PNs. Of these cases, 85 and 17 had true and false negative PNs. The baseline characteristics of these patients are presented in Table 1. Of the 85 true negative PNs, 59 and 26 were confirmed by CT and surgical resection, respectively. Of the 17 false negative PNs, 10 and 7 were respectively confirmed via repeat CTB and surgical resection.

**Table 1:** Patients baseline data

	Training cohort			Test cohort			*P* value between 2 groups
	True negative	False negative	*P*	True negative	False negative	*P*	
Patients number	85	17		50	12		
Normal data							
Age (year)	56.5 ± 12.7	64.9 ± 5.7	0.009	57.0 ± 11.0	64.2 ± 5.7	0.033	0.779
Sex (male/female)	49/36	9/8	0.721	30/20	6/6	0.528	0.880
Smoking history	35	7	1.000	25	6	1.000	0.270
Imaging feature							
PN size (mm)	18.5 ± 5.7	18.4 ± 4.9	0.912	18.3 ± 6.6	17.7 ± 3.8	0.758	0.721
SUV_max_	1.6 (Q1: 0; Q3: 2.5)	5.5 (Q1: 3.4; Q3: 8.5)	0.001	1.6 (Q1: 0; Q3: 2.4)	3.7 (Q1: 2.3; Q3: 7.5)	0.001	0.848
Right lung/Left lung	44/41	8/9	0.723	29/21	6/6	0.616	0.496
Upper lobe/Non-upper lobe	41/44	7/10	0.595	22/28	7/5	0.372	0.972
Details of biopsy procedure							
Intrapulmonary needle distance (mm)	13 (Q1: 5; Q3: 24)	17 (Q1: 6; Q3: 25.5)	0.537	14.5 (Q1: 5; Q3: 22.5)	22.5 (Q1: 11.5; Q3: 37.8)	0.058	0.718
Needle - pleura angle (degrees)	69.1 ± 19.1	67.4 ± 23.1	0.752	67.5 ± 18.6	69.7 ± 21.8	0.730	0.821
Number of specimen	3.1 ± 0.6	3.1 ± 0.6	0.830	3.0 ± 0.7	2.8 ± 0.6	0.262	0.188
Pneumothorax	13	4	0.635	6	3	0.489	0.715
Pulmonary hemorrhage	16	5	0.511	10	4	0.543	0.546
Tumor marker							
CEA (ug/L)	1.8 (Q1: 1.3; Q3: 2.7)	3.1 (Q1: 1.9; Q3: 6.4)	0.003	1.7 (Q1: 1.3; Q3: 2.4)	3.2 (Q1: 1.9; Q3: 6.2)	0.01	0.246
Cyfra21-1 (ng/ml)	2 (Q1: 1.4; Q3: 2.9)	2.2 (Q1: 1.9; Q3: 2.8)	0.210	2 (Q1: 1.4; Q3: 2.9)	2.2 (Q1: 1.8; Q3: 3.1)	0.276	0.908
SCC (ug/L)	0.8 (Q1: 0.5; Q3: 1.5)	1 (Q1: 0.6; Q3: 1.7)	0.413	0.8 (Q1: 0.5; Q3: 1.3)	0.9 (Q1: 0.7; Q3: 1.6)	0.177	0.322
NSE (ng/ml)	11.7 ± 2.6	11.9 ± 1.5	0.836	12.1 ± 2.8	12.4 ± 1.1	0.755	0.323
Pathological feature of biopsy			0.001			0.001	0.221
Chronic inflammation	27	6		7	4		
Granulomatous inflammation	44	2		32	0		
Alveolar epithelial hyperplasia	13	3		10	3		
Suspected malignant cell	1	6		2	4		

CEA: carcinoembryonic antigen; NSE: neuron-specific enolase; PN: pulmonary nodule; SCC: squamous cell carcinoma antigen; SUV_max_: standardized maximum uptake value

Relative to patients with false negative results, those with true negative results were significantly younger (*P* = 0.009). Median SUV_max_ and carcinoembryonic antigen (CEA) levels in the false negative group were significantly higher than those in the true negative group (*P* = 0.001 and 0.003). In comparison to false negative results, true negative results were also associated with a higher proportion of CTB-based granulomatous inflammation results (51.8% vs. 11.8%) and lower rates of CTB-based findings of suspected malignant cells (1.2% vs. 35.3%).

### Predictive factors of false negative

In univariate analyses, significant predictors of false negative results included older age (*P* = 0.013), higher SUV_max_ values (*P* = 0.001), higher CEA levels (*P* = 0.036), and CTB-based findings of suspected malignant cells (*P* = 0.001), whereas CTB-based findings of granulomatous inflammation (*P* = 0.008) were predictive of true negative results. Subsequent multivariate analyses revealed that the only independent predictors of false-negative outcomes were higher SUV_max_ values (*P* = 0.001) and CTB-based findings of suspected malignant cells (*P* = 0.043, Table 2).

**Table 2:** Risk factors of false-negative

	Univariate analysis			Multivariate analysis		
	Hazard ratio	95% CI	*P* value	Hazard ratio	95% CI	*P* value
Age	1.083	1.017–1.153	0.013	1.142	0.977–1.336	0.096
Gender						
Male	1					
Female	1.210	0.425–3.440	0.721			
Smoking history						
No	1					
Yes	1.000	0.347–2.881	1.000			
PN size	0.995	0.906–1.092	0.911			
Lung sides						
Right	1					
Left	1.207	0.425–3.426	0.723			
Lung lobes						
Non-upper	1					
Upper	0.751	0.261–2.158	0.595			
SUV_max_	1.926	1.443–2.570	0.001	2.361	1.444–3.859	0.001
Intrapulmonary needle distance	1.007	0.974–1.042	0.680			
Needle - pleura angle	0.996	0.970–1.022	0.750			
Number of specimen	1.099	0.469–2.577	0.828			
Pneumothorax						
No	1					
Yes	1.704	0.480–6.049	0.410			
Pulmonary hemorrhage						
No	1					
Yes	1.797	0.554–5.827	1.797			
CEA	1.140	1.009–1.287	0.036	1.085	0.858–1.372	0.498
Cyfra21-1	1.253	0.857–1.832	0.244			
SCC	1.114	0.927–1.340	0.250			
NSE	1.023	0.824–1.270	0.834			
Granulomatous inflammation	0.124	0.027–0.577	0.008	0.152	0.019–1.188	0.072
Alveolar epithelial hyperplasia	1.187	0.299–4.716	0.808			
Suspected malignant cell	45.818	5.035-416.963	0.001	35.572	1.122–112.8	0.043

CEA: carcinoembryonic antigen; NSE: neuron-specific enolase; PN: pulmonary nodule; SCC: squamous cell carcinoma antigen; SUV_max_: standardized maximum uptake value

### Predictive model

**Fig. 3:**
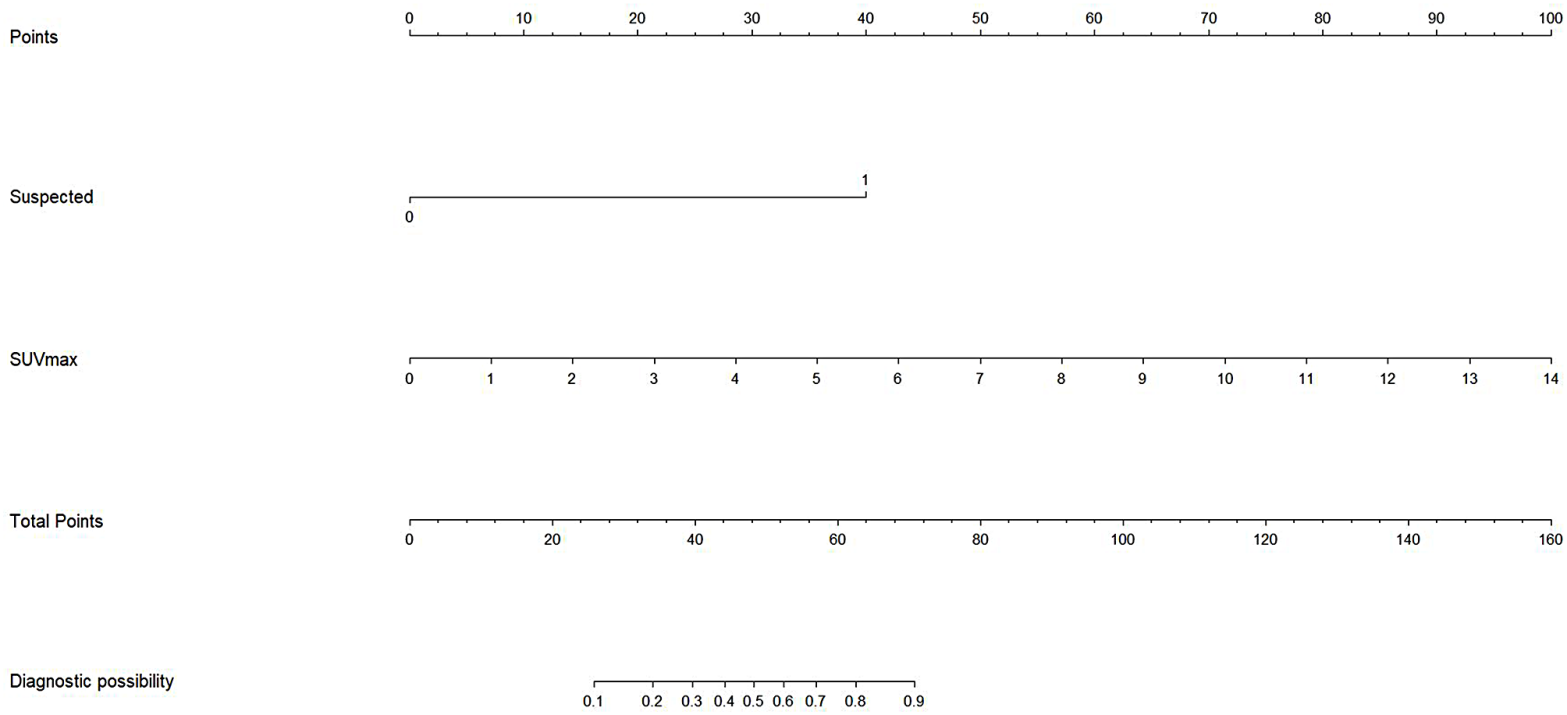
The nomogram of this predictive model

The following formula was used to construct a prediction model based on predictive factors associated with false-negative results in the aforementioned analyses: X = -13.160 + 0.859×SUV_max_ + 3.572×CTB-based finding of suspected malignant cells (yes: 1; no: 0). A cut-off risk score of -10.0247 was selected to maximize sensitivity (88.2%) and specificity (87.1%). PNs were classified as false negatives when scores were ≥ -10.0247, and as true negatives otherwise. A nomogram for the established predictive model is presented in Fig. 3.

**Fig. 4:**
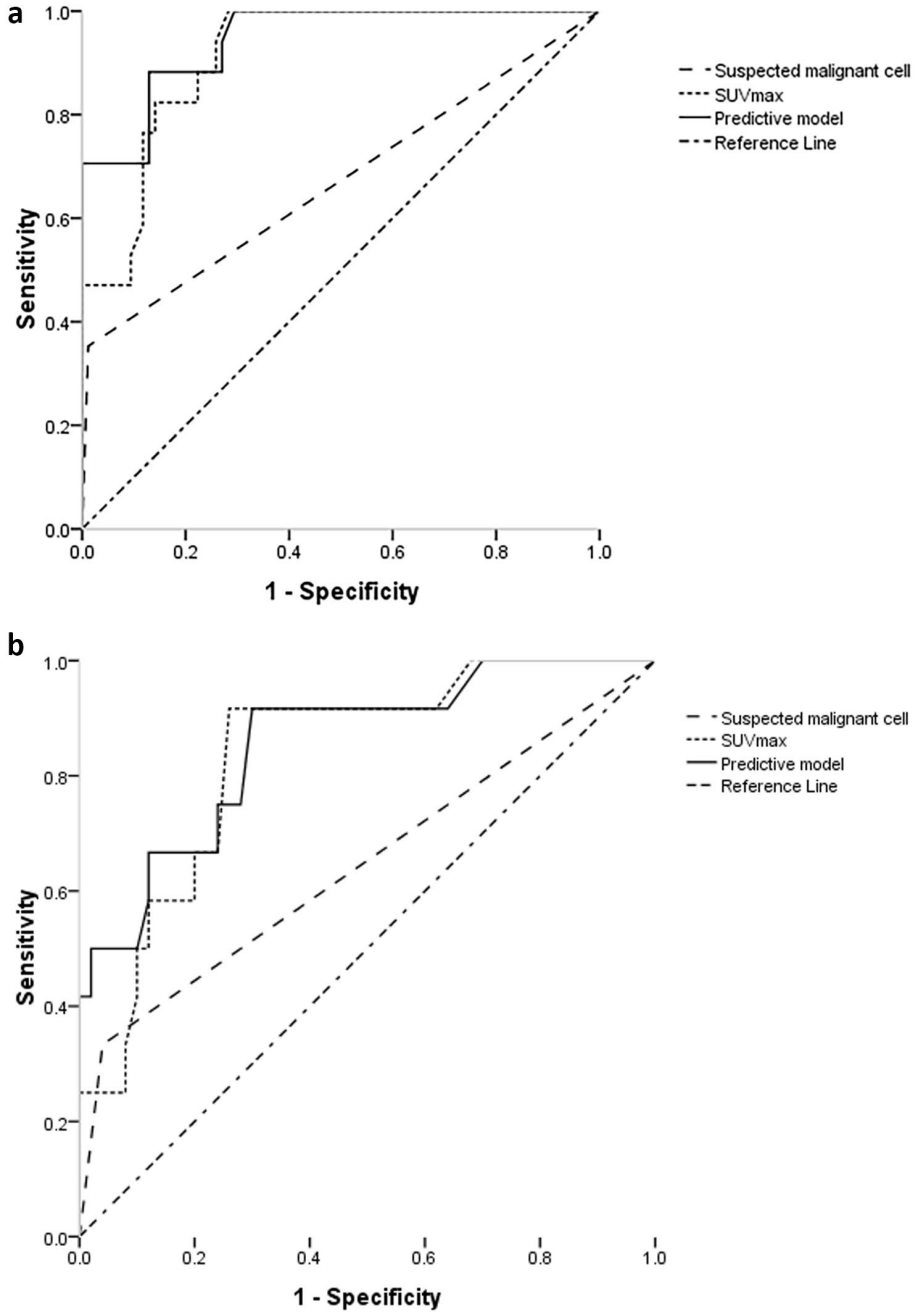
The ROC of predictive model in the (**a**) training and (**b**) test sets

ROC curves exhibited respective AUC values of 0.945, 0.915, and 0.671 for the predictive model, SUV_max_ values, and CTB-based findings of suspected malignant cells (Fig. 4a). While the former two AUC values were similar (*P* = 0.114), the AUC values for the predictive model and SUV_max_ values were significantly higher than those for CTB-based results of suspected malignant cells (both *P* = 0.001).

### Testing cohort

The testing cohort included 62 patients, each of whom had a single PN (Table 1). All PNs were solid PNs. The lesions consisted of 50 true negatives and 12 false negatives. There were no significant differences in baseline data between the training and testing cohorts. Upon including the features of these patients into the existing prediction model, the corresponding AUC values for this predictive model, SUV_max_ values, and CTB-based findings of suspected malignant cells were determined to be 0.851, 0.834, and 0.647, respectively (Fig. 4b). The AUC values for the predictive model and SUV_max_ values were significantly higher than those for CTB-based findings of suspected malignant cells (*P* = 0.003 and 0.031), while the former two AUC values were similar (*P* = 0.461).

### Assessment of model utility in the clinic

**Fig. 5:**
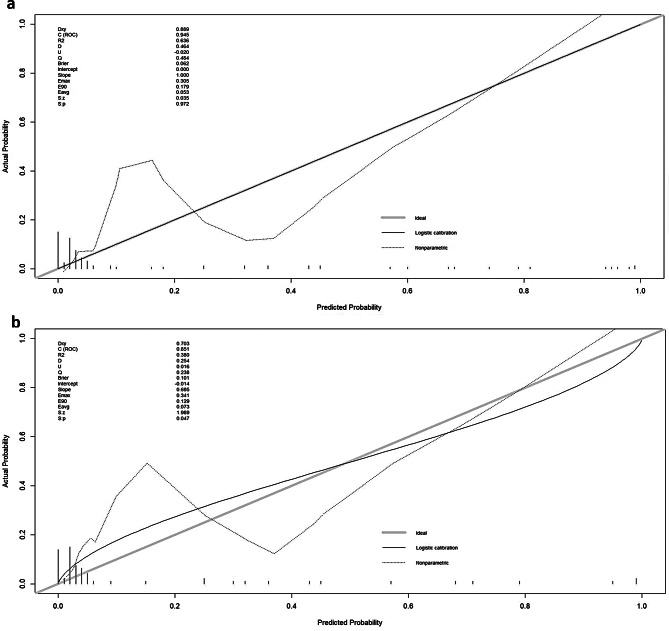
The calibration curve of predictive model in the (**a**) training and (**b**) test sets

**Fig. 6:**
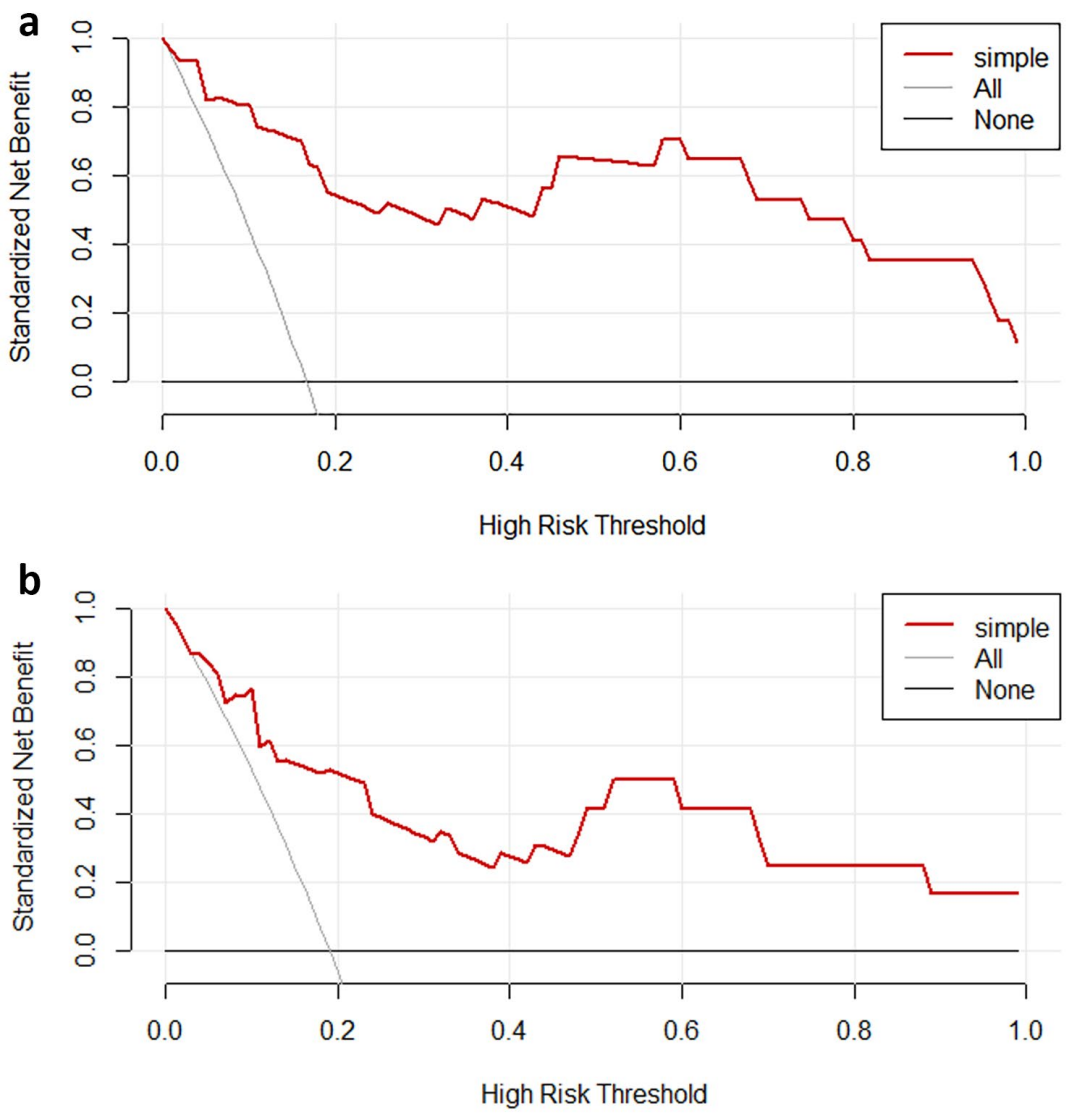
The decision curve of predictive model in the (**a**) training and (**b**) test sets

For both the training and testing cohorts, calibration curve analysis showed good consistency between expected and actual false negative results (Fig. 5). With matching risk criteria of 0–1 and 0–1, a decision curve analysis for this model demonstrated considerable net benefits in both patient cohorts (Fig. 6).

## Discussion

In the present study, the rate of correctly identifying individuals without PNs as negative was 83.3%. This rate is consistent with rates reported in previous publications (83.6–89.4%) [4–6]. These findings indicate that CTB-based non-malignant pathology results tend to be reliable.

The confirmation of true or false negative CTB-based results usually requires either a prolonged period of monitoring or intrusive interventions, such as surgery or repeat biopsy [4–6]. Accurately predicting the false negative status might significantly influence clinical decision-making in cases when CTB-based results indicate non-malignancy.

The CTB-based detection of granulomatous inflammation has previously been reported as a valuable predictive factor when identifying true negative results for patients with CTB-based non-malignant findings [4–6]. The formation of a granuloma can occur in response to chronic infections as a form of protective response, and it can also be engaged in response to certain non-infectious stimuli [11, 12]. Granulomatous lung disease may be caused by infection, autoimmunity, aspiration pneumonia, hypersensitivity pneumonitis, sarcoidosis, and talc granulomatosis [13]. Granulomas have been observed to be associated with malignant tumors only infrequently [14]. In the training cohort, 2 (4%) of 46 cases identified as instances of CTB-based granulomatous inflammation were false negatives. Despite this low rate, however, CTB-based detection of granulomatous inflammation was not significantly predictive of true negative results in the present study. This may be because 51.8% (44/85) of the true negative cases exhibited CTB-based granulomatous inflammation.

The results of this study were consistent with those of similar previous studies that focused on false negative outcomes for lung lesions following an initial biopsy-based non-malignant pathological result. CTB-based findings of suspected malignant cells were identified as a risk factor significantly associated with false negative results [15, 16]. However, the AUC values in the training and assessment cohorts were only 0.671 and 0.647 when evaluating the ROC curves for CTB-based findings of suspected malignant cells. The reason for this may be that this is a categorical variable, and/or because just 7 cases in the training cohort presented with CTB-based findings of suspected malignant cells.

^18^F-fludeoxyglucose (^18^F-FDG) PET/CT imaging strategies can generate CT images that highlight morphological details of a particular lesion together with PET images that reveal the metabolic activity of these lesions. Malignant lesions usually display elevated glucose levels and increased absorption of ^18^F-FDG compared to neighboring non-malignant cells due to their heightened metabolic activity [17]. In this study, PET/CT images were found to be highly effective in accurately differentiating between true and false negative CTB-based results. The diagnostic utility of PET/CT was demonstrated by the respective AUC values of 0.915 and 0.834, which were assessed in the training and testing cohorts. PET/CT imaging yielded AUC values that were significantly higher than those associated with CTB-based findings of suspected malignant cells in both of these patient cohorts (*P* = 0.001 and 0.031). Analyzing PET/CT-derived SUV_max_ values may thus offer greater diagnostic utility than relying on CTB-based findings of suspected malignant cells.

Although PET/CT has the great diagnostic significance in the PNs, some non-represented cases still exist. For example, granulomatous inflammation and chronic inflammation can lead to increased SUV, while some malignant tumors (including well-differentiated adenocarcinoma, keratinizing squamous cell carcinoma, carcinoid, etc.) exhibit low SUV. Therefore, the relative predictors of false-negative and true-negative should be analyzed simultaneously when managing the non-represented cases. To combined these predictors rationally, the predictors identified in this study were used to develop a predictive model according to the hazard ratio, and a corresponding nomogram was also established. The resultant model was able to produce a comprehensive diagnostic score corresponding to a given lesion of interest, while the nomogram can use this score to determine the odds of that lesion being malignant. In the training cohort, this model exhibited an AUC of 0.945 with corresponding sensitivity and specificity values of 88.2% and 87.1%. These results, coupled with the AUC of 0.851 measured in the testing cohort. Although the AUC values in the training and testing cohorts are different, both the AUC values are larger than 0.8. These findings may demonstrated the accuracy of this model. However, the stability of this model should be further validated by more data from other centers.

There are certain limitations to these analyses. Due to the retrospective nature of this study, there is a significant possibility of selection bias, making it crucial to do prospective validation. In addition, the absence of final diagnoses in some cases necessitated the exclusion of specific patients from this study, potentially resulting in a discrepancy between the calculated true negative rate and the actual rate. Furthermore, the SUV_max_ value may vary largely due to physical and biological factor, different types of PET/CT device, and the operators’ experience. This point may further increase the selection bias. However, in this study, the 2 centers used the same type of PET/CT device and the scanning protocols were not change during the study period. These may decrease the risk of bias. Finally, a logistic regression approach was employed in the design of the predictive model used in this study. However, probably, the utilization of machine learning models such as random forest, k-nearest neighbors, XG boost, support vector machine, or light GBM approaches could have resulted in more accurate predictive outcomes.

## Conclusions

In summary, in patients with PNs, both a higher PET/CT SUV_max_ value and CTB-based findings of suspected malignant cells are indicative of a higher risk of false negative CTB-based non-malignant pathological results. These predictive factors were effectively utilized to develop a predictive model that demonstrated good diagnostic accuracy in detecting false negative results in patients with a CTB-based non-malignant result.

## Data Availability

No datasets were generated or analysed during the current study.

## Abbreviations

AUC: Area under curve
CEA: Carcinoembryonic antigen
CT: Computed tomography
CTB: CT-guided biopsy
NSE: Neuron-specific enolase
PET: Positron emission tomography
PN: Pulmonary nodule
ROC: Receiver operator characteristic
SCC: Squamous cell carcinoma antigen
SUV_max_: Standardized maximum uptake value
